# Serum Vascular Endothelial Growth Factor Levels Correlate with Severity of Retinopathy in Diabetic Patients: A Systematic Review and Meta-Analysis

**DOI:** 10.1155/2019/9401628

**Published:** 2019-03-24

**Authors:** Zhongwei Zhou, Huixiang Ju, Mingzhong Sun, Hongmei Chen

**Affiliations:** Department of Clinical Laboratory, Affiliated Yancheng Hospital, School of Medicine, Southeast University, Yancheng, Jiangsu 224001, China

## Abstract

**Background:**

Investigations regarding serum and plasma vascular endothelial growth factor (VEGF) levels in patients with diabetic retinopathy (DR) are conflicting. This meta-analysis is aimed at determining whether serum and plasma VEGF levels are associated with DR and its severity in diabetic patients.

**Methods:**

PubMed and EMBASE were used to search for published studies, and serum and plasma VEGF levels were compared among DR, nonproliferative diabetic retinopathy (NPDR), proliferative diabetic retinopathy (PDR), and nondiabetic retinopathy (NDR) patients. Standardized mean differences (SMD) and 95% confidence interval (CI) were pooled using a random effects model.

**Results:**

A total of 29 studies comprising 1805 DR (or NPDR or PDR) patients and 1699 NDR patients were included. ELISA was used to evaluate serum or plasma VEGF levels in all except for two studies included in this meta-analysis. Overall, serum VEGF levels were significantly higher in DR patients (SMD: 0.74, 95% CI: 0.44-1.03) than those in NDR patients, while plasma VEGF levels were not in the comparison (SMD: 0.40, 95% CI: −0.13-0.92). Similarly, NPDR (SMD: 0.51, 95% CI: 0.22-0.80) and PDR (SMD: 1.32, 95% CI: 0.79-1.85) patients had higher serum VEGF levels compared with NDR patients, but the difference was not significant in plasma samples (SMD: 0.24, 95% CI: −0.47-0.95; SMD: 0.37, 95% CI: −0.30-1.05). In addition, serum VEGF levels were higher in PDR patients than those in NPDR patients (SMD: 0.87, 95% CI: 0.41-1.33), but plasma VEGF levels were not (SMD: −0.00, 95% CI: −0.31-0.31). The subgroup and metaregression analysis revealed that the study location, study design, and publication year of a study have certain influence on heterogeneity between studies in serum or plasma samples.

**Conclusions:**

VEGF levels in the serum instead of those in the plasma correlate to the presence and severity of DR in diabetic patients. Further large-scale studies are required to confirm these findings.

## 1. Introduction

Diabetic retinopathy (DR) is one of the most common microvascular complications of diabetes mellitus, and it can be clinically classified into nonproliferative diabetic retinopathy (NPDR) and proliferative diabetic retinopathy (PDR), depending on whether or not neovascularization is found in the retina [1, 2].

The pathogenesis of DR is extremely complicated. The regulating process involves multiple retinal cells such as retinal astrocytes, endothelial, Muller, and pigment epithelium cells, and vascular endothelial growth factors (VEGF) is expressed in all of the above cells [3–5]. VEGF is the most potent vasoactive factor, the normal expression of which is necessary for maintaining the structural and functional homeostasis of the retinal cells, but whose overexpression could lead to retinal angiogenesis in the effects of pathological factors such as hypoxia and hyperglycemia [6]. In diabetic rat models, retinal angiogenesis occurred at about six months, and at the same time, VEGF was significantly highly expressed in both retinal tissue and serum [7]; and the change dynamics of VEGF expression in serum were remarkably similar to those in the retina and vitreous with the progression of DR [8]. Furthermore, clinical studies showed that vitreous and circulating VEGF in the serum or plasma was increased markedly in patients with PDR, and there was a significantly positive correlation between them [9, 10]. In addition, when bevacizumab, an angiogenesis inhibitor, was injected into the vitreous body of PDR patients, VEGF expression in the serum/plasma, aqueous and vitreous, was significantly decreased [11, 12]. Therefore, VEGF is a good biomarker for evaluating the progression and therapeutic effects of DR. But as far as disease markers are concerned, ocular fluids are hard to be used widely due to their poor collection and greater pain to patients. By contrast, serum or plasma has many advantages in the assessment of the development and prognosis of the diseases, for example, accessibility, noninvasiveness, and easy to continuous monitoring [13].

However, studies on the association of serum and plasma VEGF levels with DR and its severity are inconsistent. Therefore, we performed a meta-analysis to provide a more comprehensive conclusion of the association of serum and plasma VEGF levels with DR and its severity in diabetic patients.

## 2. Materials and Methods

### 2.1. Search Strategy

A systematic literature search was carried out in electronic databases including PubMed and EMBASE up to May 2018. The search terms included (“diabetic retinopathy” OR “nonproliferative diabetic retinopathy” OR “proliferative diabetic retinopathy” OR “DR” OR “NPDR” OR “PDR”) AND (“vascular endothelial growth factors” OR “VEGF”). In addition, the reference list of the selected articles was manually searched for additional eligible studies.

### 2.2. Eligibility Criteria

Studies reporting serum and plasma VEGF levels in DR patients were eligible for review. The additional inclusion criteria were (1) studies in adult subjects (age ≥ 18 years), (2) the study population of diabetic patients, (3) DR (or NPDR or PDR) which was the outcome and the control group consisting of nondiabetic retinopathy (NDR) patients, or (4) the study published in English. The exclusion criteria were as follows: (1) studies that examined pregnancy associated with diabetes, (2) studies that were interventional with similar groups at baseline, (3) samples that overlapped with another study, or (4) review article, case reports, letters to the editor, conference abstracts, or in vitro studies.

### 2.3. Data Extraction and Quality Assessment

Two investigators independently extracted data from the included studies and confirmed by a third reviewer. Disagreement was resolved by discussion among all researchers. The following information was abstracted from each eligible study: the first author's name, year of publication, country of data collection, study design, the assay method of VEGF, diabetes type, sample source, clinical characteristics of patients (age, gender, BMI), and mean and standard deviation (SD) of VEGF levels in the case and control group. If studies provided ranges or interquartile ranges instead of the mean and SD, transformations were made by formulas which were proposed by Higgins et al. [14] and Hozo et al. [15]. The unit of VEGF measurement was uniformly converted to pg/mL in this meta-analysis.

The quality of the study was evaluated using a modified criteria based on the Newcastle-Ottawa Quality Assessment Scale (NOS) for observational studies suggested by van Dijk et al. [16]. The scale included the assessment of three overall domains: selection, comparability, and exposure. The full score was 9 stars, and a study that met 7 or more stars would be considered a high-quality study and less than 3 stars a low-quality study, and other studies were defined as moderate quality.

### 2.4. Statistical Analysis

We used Stata 15.0 (StataCorp LP, College Station, TX, USA) to perform all statistical analyses. To compare VEGF levels between patients with DR (or NPDR or PDR) and the NDR population, pooled analyses were performed using standardized mean differences (SMD) and its corresponding 95% confidence interval (CI). We used a random effects model which would be more conservative than the fixed effects model to calculate the pooled estimate [17], because within-study and between-study confounders might account for the anticipated heterogeneity. The *I*^2^ test was used to assess the significance of heterogeneity among studies, and an *I*^2^ index of 25%, 50%, and 75% would indicate small, moderate, and high heterogeneity, respectively [18]. To explore the potential moderating effects of continuous variables on the pooled outcome, metaregression analysis was carried out. We assumed the publication year, mean age, BMI, and the number of patients and patient sex as potential moderators for the outcome of the meta-analysis.

Sensitivity analysis was undertaken to evaluate whether the pooled measures were influenced by a single study by removing one study at a time and recalculating the pooled SMD for the remainders. Publication bias was evaluated by inspection of funnel plots and Egger's test.

Publication bias was first visually inspected by funnel plots, and the statistical significance was determined by Egger's test.

*P* < 0.05 was considered to be statistically significant.

## 3. Results

### 3.1. Literature Search

**Figure 1:**
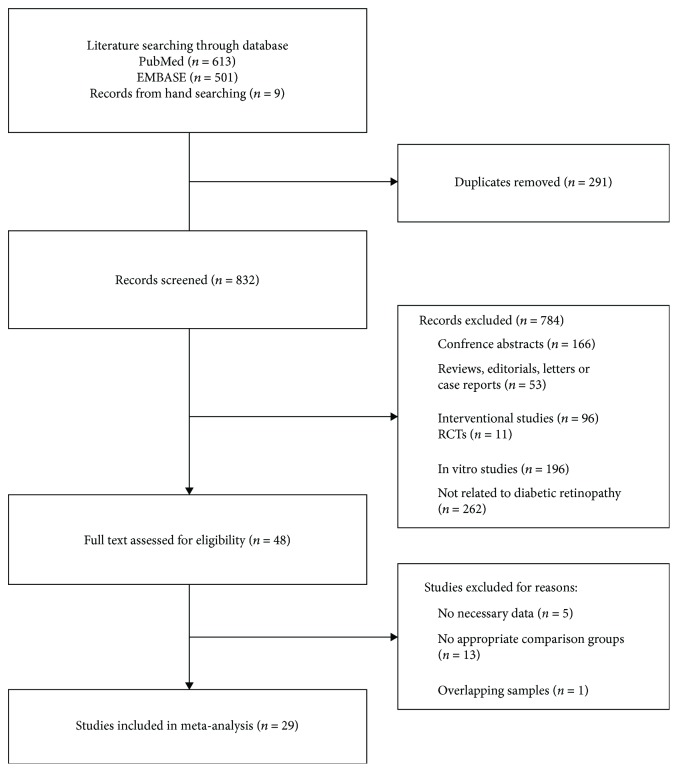
Flow chart of the study selection process.

We first performed a systematic search, which yielded 613 records from PubMed and 501 records from EMBASE, and 9 additional records were identified by searching the reference lists of selected articles. After reading the titles and abstracts, 48 appropriate articles were identified for full-text analysis. The 19 articles were further excluded for lack of necessary data on VEGF levels, no appropriate comparison groups, and patient samples that overlapped with another study. Finally, 29 studies met the inclusion criteria [19–47], and a flowchart of the included and excluded studies is shown in Figure 1.

### 3.2. Characteristics of the Included Studies

The main characteristics of the included studies are summarized in Table 1. The 29 included studies were published from 1997 to 2017 covering 1805 DR (or NPDR or PDR) patients and 1699 NDR patients in 13 countries. Among these, 19 studies investigated serum VEGF levels and 10 plasma VEGF levels; 20 studies were cross-sectional, 7 case-control, and 1 cohort design. The patients in 25 studies were type 2 diabetes, two type 1 diabetes, and one both type 1 and 2. The patient number of these studies ranged from 10 to 372, and the range of the mean VEGF levels was 13.05 to 775.13 pg/mL. The age, gender, and BMI of DR patients were reported in twenty-two, seventeen, and twelve studies, respectively. DR patients were classified into NPDR and PDR in twenty studies, while five studies did not provide the categories of DR, and three included only PDR and one NPDR.

A quality score was evaluated across these included studies. Fourteen studies were scored greater than or equal to 7 out of 9 which were considered high-quality studies, and the other fifteen studies were evaluated as moderate quality. No studies were assessed as low-quality; however, 2 studies, by Skopiński et al. [42] and Shimada et al. [44], were graded 3.

### 3.3. Meta-analysis

**Figure 2:**
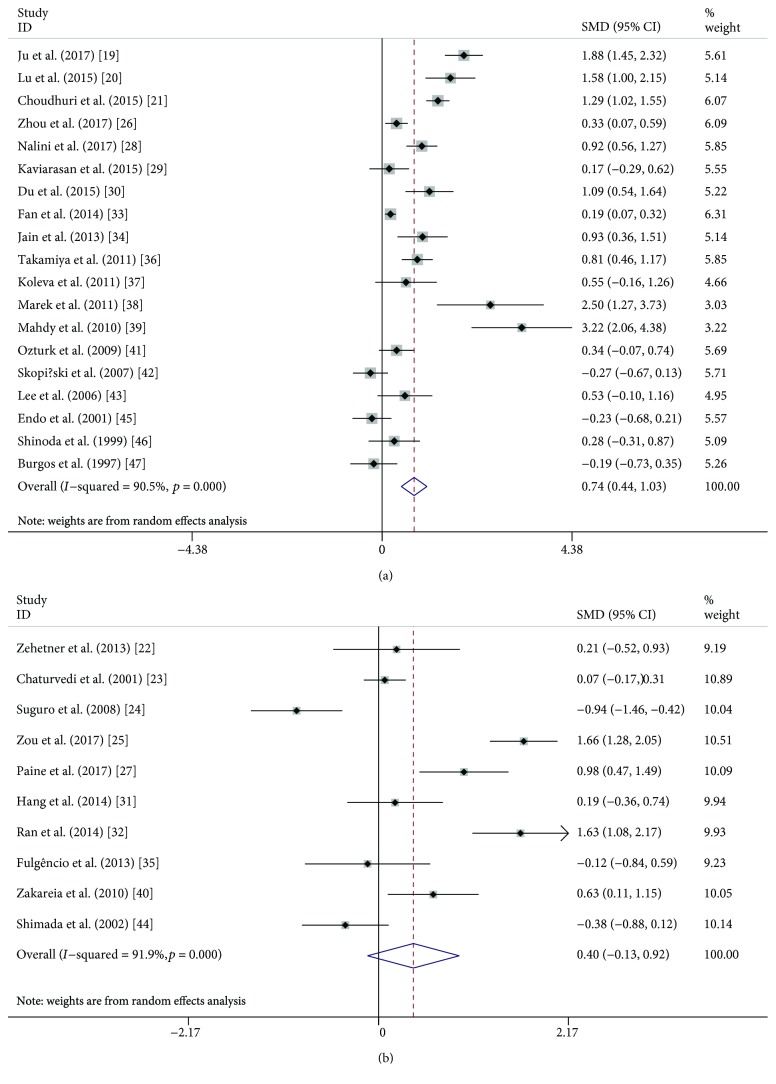
Forest plot summarizing the relationship of serum and plasma VEGF level in DR patients with those in NDR patients: serum (a) and plasma (b).

**Figure 3:**
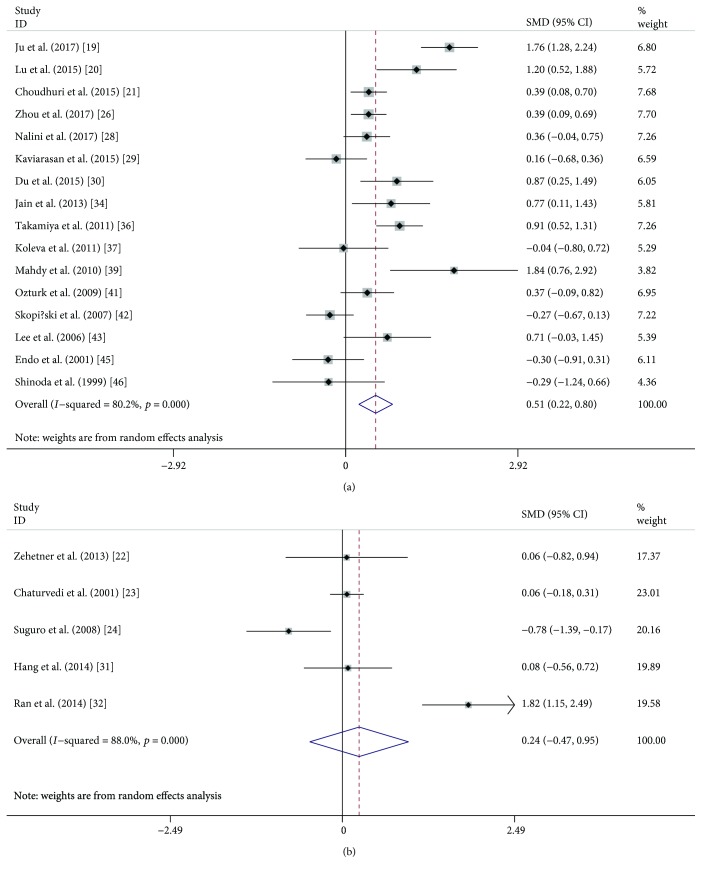
Forest plot summarizing the relationship of serum and plasma VEGF level in NPDR patients with those in NDR patients: serum (a) and plasma (b).

**Figure 4:**
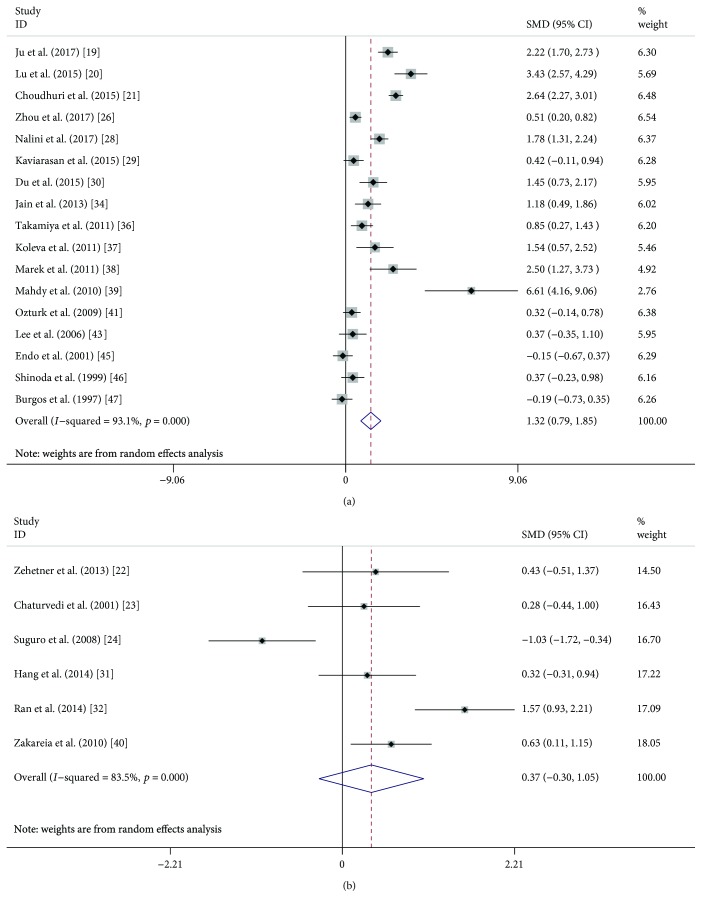
Forest plot summarizing the relationship of serum and plasma VEGF level in PDR patients with those in NDR patients: serum (a) and plasma (b).

**Figure 5:**
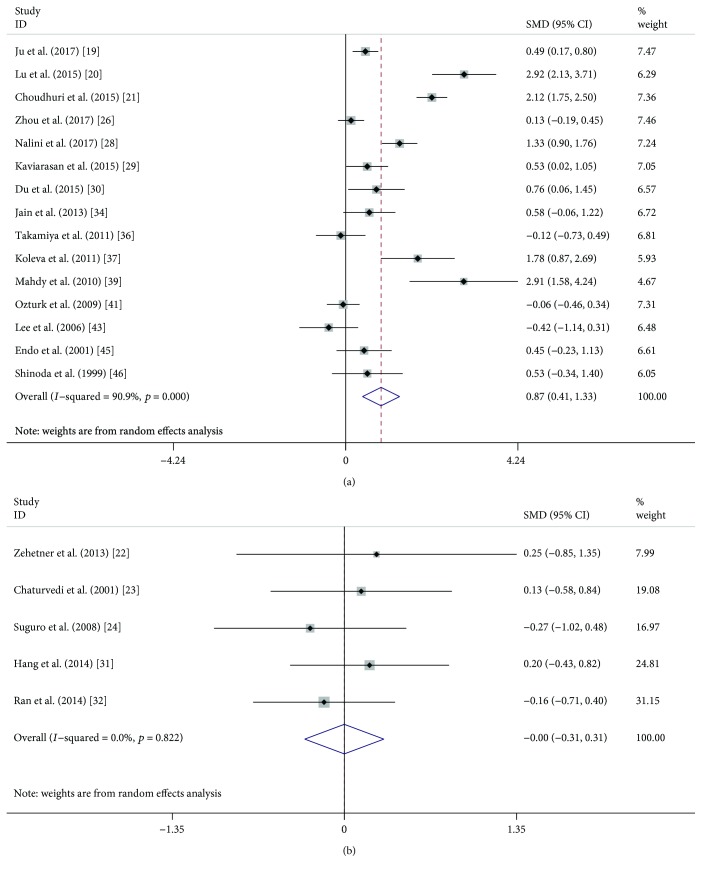
Forest plot summarizing the relationship of serum and plasma VEGF level in PDR patients with those in NPDR patients: serum (a) and plasma (b).

A random effects meta-analysis was performed, and serum and plasma VEGF levels were compared between DR (or NPDR or PDR) and NDR patients. Overall, serum VEGF levels were significantly higher in DR patients (SMD: 0.74, 95% CI: 0.44-1.03, *P* < 0.001) than those in NDR patients (Figure 2(a)), while plasma VEGF levels did not show a significant difference in the comparison (SMD: 0.40, 95% CI: −0.13-0.92, *P* = 0.136) (Figure 2(b)). When DR patients were classified into NPDR and PDR, both NPDR (SMD: 0.51, 95% CI: 0.22-0.80, *P* < 0.001) and PDR (SMD: 1.32, 95% CI: 0.79-1.85, *P* < 0.001) patients had significantly higher serum VEGF levels compared with NDR patients (Figures 3(a) and 4(a)), but the difference was also not observed in plasma samples (SMD: 0.24, 95% CI: −0.47-0.95, *P* = 0.507; SMD: 0.37, 95% CI: −0.30-1.05, *P* = 0.279) (Figures 3(b) and 4(b)). Similarly, serum VEGF levels were higher in PDR patients than those in NPDR patients (SMD: 0.87, 95% CI: 0.41-1.33, *P* < 0.001) (Figure 5(a)), but plasma VEGF levels were not (SMD: −0.00, 95% CI: −0.31-0.31, *P* = 0.994) (Figure 5(b)). High levels of heterogeneity among studies were found in all these comparisons (*I*^2^ ranged from 80.2% to 93.1%) except the comparison of PDR vs. NPDR in the plasma sample (*I*^2^ = 0.0%, *P* = 0.822).

### 3.4. Subgroup Analysis

Subgroup analysis was performed based on the study location and study design, which are shown in Table 2. When the studies were stratified according to the study location, Asian and African patients with DR had significantly higher serum VEGF levels compared with NDR patients (*P* < 0.001), while serum VEGF levels in European patients and plasma VEGF levels in Asian, African, and South American patients did not show a significant difference in the comparison of DR vs. NDR (*P* > 0.05). Although no heterogeneity was observed in the plasma samples of European patients (*P* > 0.05), considerable heterogeneity was still found in the serum samples of Asian and European patients and in the plasma samples of Asian patients (*P* < 0.001). When stratifying by the study design, the subgroups of cross-sectional and cohort study showed higher serum VEGF levels (*P* < 0.001), while serum VEGF levels in the case-control subgroup and plasma VEGF levels in all these subgroups did not show a significant difference in the comparison of DR vs. NDR (*P* > 0.05). Significantly decreased heterogeneity was observed in the serum samples of the case-control study (*P* > 0.05); however, significant heterogeneity was still found in both serum and plasma samples of the cross-sectional study (*P* < 0.001).

### 3.5. Metaregression Analysis

**Figure 6:**
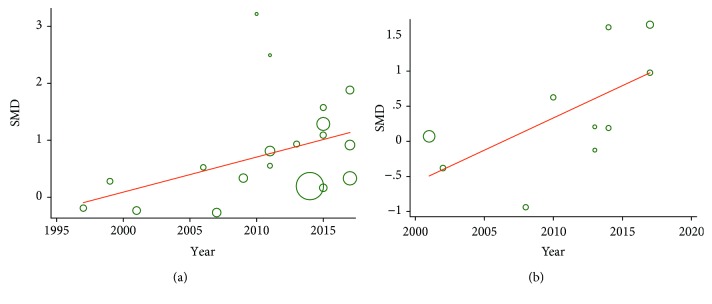
Metaregression analysis of the relationship between the publication year and the effect sizes in serum and plasma samples: serum (a) and plasma (b).

To investigate whether the continuous variables, including the publication year of each study, mean age, BMI, and the number of DR patients (males), had potential moderating effects on the pooled SMD, a random effects metaregression analysis was performed. We found there was a weak positive correlation between the publication year and the effect sizes in both serum (*β* = 1.064, *P* = 0.052; Figure 6(a)) and plasma samples (*β* = 1.096, *P* = 0.051; Figure 6(b)), while other tested variables did not show moderating effects on the pooled outcome in studies involving these variables (*P* > 0.05).

### 3.6. Sensitivity Analysis

**Figure 7:**
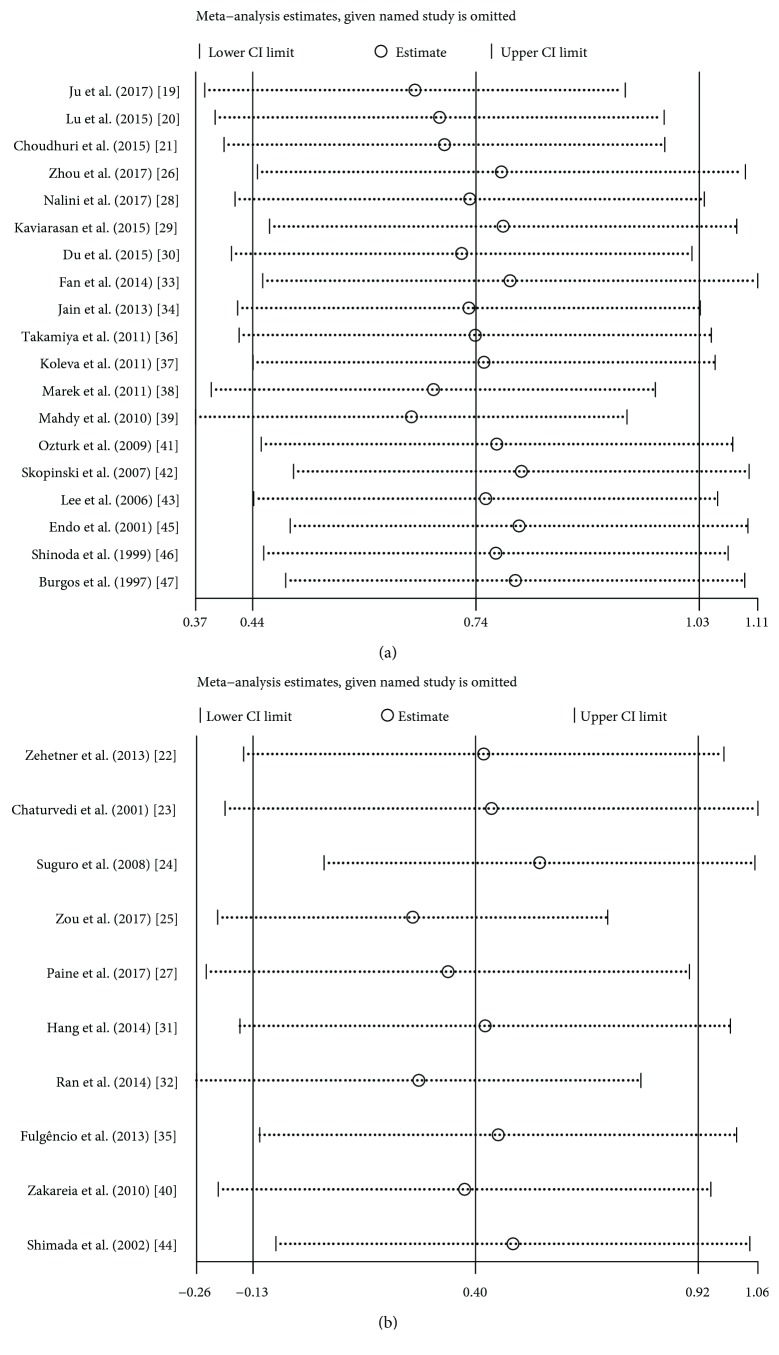
Sensitivity analysis of included studies: serum (a) and plasma (b).

In the sensitivity analysis, we found that no individual study significantly influenced the difference on VEGF levels in both serum (Figure 7(a)) and plasma (Figure 7(b)) samples.

### 3.7. Publication Bias

**Figure 8:**
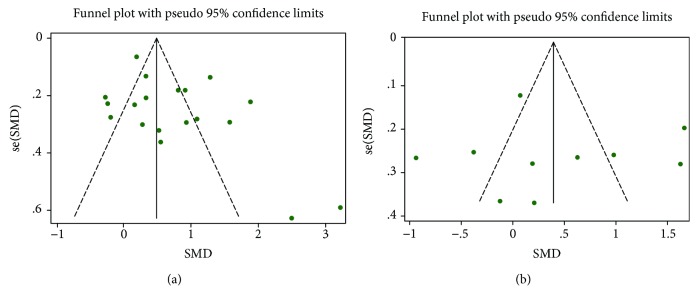
Visual inspection of funnel plots evaluating potential publication bias of included studies: serum (a) and plasma (b).

Visual inspection of funnel plots showed that no sign of publication bias was observed in both serum (Figure 8(a)) and plasma (Figure 8(b)) samples in this meta-analysis, and the results were further confirmed by Egger's test (*P* = 0.688 and *P* = 0.729).

## 4. Discussion

In this study, we performed the meta-analysis separately in serum and plasma to determine whether VEGF levels were associated with DR and its severity in diabetic patients. We found that serum VEGF levels in DR, NPDR, and PDR patients were significantly higher than those in NDR patients and PDR patients than NPDR patients, but these differences were not found in plasma samples. Further stratified analyses showed higher serum VEGF levels in DR patients were especially available in the subgroups of Asian population and cross-sectional study. Metaregression analysis demonstrated the publication year was positively associated with the effect sizes. Sensitivity analysis indicated these findings were not essentially influenced by any single study, and no significant publication bias was observed in the meta-analysis of both sample types.

We did not find the significant difference of serum VEGF levels between DR and NDR patients in the subgroups of European patients and case-control study, which indicates there may be varying serum VEGF levels in DR patients with different ethnic backgrounds, and study design might influence the results of VEGF expression in serum. But an alternative explanation may be the result of fewer studies included in these subgroups. However, it should be emphasized that plasma VEGF levels did not show a significant difference in all subgroups based on a stratified study location and study design, which further strengthens the conception that plasma VEGF levels may not be a sensitive indicator for evaluating the development and progression of DR. In this meta-analysis, we found a large amount of heterogeneity among studies, but the strength of this work is that subgroup analyses and metaregression analyses were used to adjust for potential confounders. In stratified analyses based on the study location and study design, we found heterogeneity disappeared in the plasma samples of European patients and markedly decreased in the serum samples of the case-control study. In metaregression analyses, we found there was a weak positive correlation between the publication year and the effect sizes in both sample types. These findings suggest the study location, study design, and publication year of study, to some extent, may explain heterogeneity between studies in serum or plasma samples.

There have been controversial views on the optimal specimen, serum, or plasma VEGF in clinical usefulness. Lee et al. [48] reported that serum was the more suitable specimen for the measurement of circulating VEGF in determining the prognosis of cancer patients, while a systematic review performed by Botelho et al. [49] pointed out the VEGF levels in the plasma instead of those in the serum were useful for differentiating benign from malignant prostatic disease. The difference between the plasma and serum is that the former uses anticoagulants to keep blood samples from clotting. It has been well known that platelets are a rich source of VEGF which is released upon their activation during clotting, which is exactly the reason for higher VEGF levels in serum samples than in matched plasma samples [48, 50]. Platelet activation has been shown to be involved in the pathogenesis and development of DR [51]. Previous studies have also shown that the mean platelet volume (MPV), which is an indicator of platelet activation, was increased progressively with the progression of DR [52, 53]. And the more the degree platelets are activated, the more the VEGF is released. Therefore, the positive correlation between VEGF levels and severity of DR in serum samples may be the result that platelets are differentially activated, and there was no significant difference between plasma VEGF levels and the progression of DR may be because of few or no platelet activation.

The pathophysiologic mechanism for increased VEGF expression involving in the development and progression of DR is not yet fully elucidated. However, several plausible explanations may account for their links. First, overexpression of VEGF induced by persistent hyperglycemia can lead to increasing vascular endothelium permeability, decreasing inhibition of proapoptotic proteins, disruption of the vascular homeostasis, and success by neovascularization in the retina [54]. Second, increasing evidence indicates inflammation is a key player in the development of DR [55] and VEGF is a strong inducer of inflammation [56]. There is also evidence that Müller cell-derived VEGF plays an essential and causative role in retinal inflammation [57]. Therefore, overexpression of VEGF exacerbates inflammatory reaction which might be responsible for the progression of DR. Finally, it is well known that matrix metalloproteinases (MMPs) are one of the major culprits in leading to DR, which cause extracellular matrix remodeling and induce retinal cell apoptosis in the retina [58]. Recent research showed that there is just an interaction between VEGF and MMPs, and VEGF is able to induce MMP expression to promote retinal neovascularization [59, 60]. Therefore, retinal damage caused by MMPs is linked to overexpression of VEGF.

Several limitations in this met-analysis should be of concern. First, all the included studies in this meta-analysis were observational, and although the serum VEGF levels may be a reflection of platelet activation, a causal link between serum or plasma VEGF levels and the presence and severity of DR in diabetic patients cannot be established. Second, the numbers of studies that analyzed plasma VEGF levels were small, especially in comparisons of NPDR vs. NDR, PDR vs. NDR, and PDR vs. NPDR patients. Therefore, further large-scale studies in plasma samples are necessary to substantiate this idea. Third, some other potential factors such as HOMA-IR and lifestyle are limited in the eligible studies included in the meta-analysis, which prevented us from further analyzing whether these confounders had moderating effects on the outcome of this meta-analysis. Finally, selective bias was probably inevitable, as only published studies in English in the selected databases were included.

## 5. Conclusions

In conclusion, we observed that VEGF levels in the serum instead of those in the plasma correlate to the presence and severity of DR in diabetic patients, which suggests serum VEGF levels are a reliable biomarker for evaluating the development and progression of DR. Further studies are necessary to confirm these findings, especially for the association between plasma VEGF levels and DR and its severity.

## Figures and Tables

**Table 1:** Main characteristics of the studies included in this meta-analysis.

Author	Year	Country	Study design	VEGF assay method	VEGF levels (pg/mL)		Age of patients (years)	BMI of patients (kg/m^2^)	Patient number (males)	Categories of DR		Diabetes type	Sample source	Quality score
					Case	Control				NPDR (number)	PDR (number)			
Ju et al. [19]	2017	China	Cross-sectional	ELISA	157.60 ± 38.0	88.97 ± 24.86	62.05	23.2	160 (78)	80	80	Type 2	Serum	7
Lu et al. [20]	2015	China	Cross-sectional	ELISA	92.29 ± 27.90	53.66 ± 7.15	53.56	24.4	55 (25)	20	35	Type 2	Serum	6
Choudhuri et al. [21]	2015	India	Cross-sectional	ELISA	195.67 ± 81.39	106.32 ± 40.61	52.84	25.72	175 (96)	70	105	Type 2	Serum	8
Zehetner et al. [22]	2013	Austria	Cross-sectional	ELISA	38.85 ± 37.57	31.71 ± 30.28	63.9	na	13 (9)	7	6	Type 2	Serum	6
Chaturvedi et al. [23]	2001	UK	Cohort	ELISA	13.05 ± 24.16	11.50 ± 16.22	na	na	175 (na)	167	8	Type 1	Plasma	7
Suguro et al. [24]	2008	Japan	Cross-sectional	ELISA	247.3 ± 465.8	812.0 ± 113.0	60.7	24.3	28 (15)	16	12	Type 2	Plasma	9
Zou et al. [25]	2017	China	Cross-sectional	ELISA	105.64 ± 12.32	87.95 ± 8.12	48.33	21.75	75 (41)	na	na	Type 2	Plasma	7
Zhou et al. [26]	2017	China	Cross-sectional	ELISA	277.52 ± 135.24	233.15 ± 129.21	59.67	25.65	150 (71)	78	72	Type 2	Serum	8
Paine et al. [27]	2017	India	Cross-sectional	ELISA	208.5 ± 93.23	123.6 ± 45.09	57.6	na	81 (60)	na	na	Type 2	Plasma	8
Nalini et al. [28]	2017	India	Cross-sectional	ELISA	98.51 ± 14.76	84.91 ± 14.87	na	na	100 (na)	50	50	Type 2	Serum	5
Kaviarasan et al. [29]	2015	India	Case-control	ELISA	775.13 ± 770.20	660.41 ± 446.25	55.0	24.6	60 (na)	30	30	Type 2	Serum	9
Du et al. [30]	2015	China	Cross-sectional	ELISA	141.33 ± 32.45	106.62 ± 29.80	56.36	25.38	35 (21)	20	15	Type 2	Serum	7
Hang et al. [31]	2014	China	Cross-sectional	Others	65.9 ± 81.0	52.2 ± 43.2	60.7	23.6	40 (14)	19	21	Type 2	Plasma	7
Ran et al. [32]	2014	China	Cross-sectional	ELISA	56.51 ± 11.60	39.00 ± 8.43	62.42	na	50 (na)	25	25	Type 2	Plasma	7
Fan et al. [33]	2014	China	Cross-sectional	ELISA	146.2 ± 143.7	121.6 ± 116.1	63.39	24.48	372 (146)	na	na	Type 2	Serum	7
Jain et al. [34]	2013	India	Cross-sectional	ELISA	357.1 ± 168.8	210.7 ± 120.2	55.23	na	39 (26)	19	20	Type 2	Serum	6
Fulgencio et al. [35]	2013	Brazil	Cross-sectional	ELISA	31.0 ± 9.2	31.9 ± 3.8	na	na	15 (na)	na	na	Type 2	Plasma	4
Takamiya et al. [36]	2011	Japan	Cross-sectional	ELISA	184.0 ± 105.3	106.5 ± 87.78	53.0	24.7	54 (37)	40	14	Type 2	Serum	6
Koleva et al. [37]	2011	Bulgaria	Cross-sectional	ELISA	301.48 ± 224.18	185.89 ± 142.0	na	na	28 (na)	17	11	Type 2	Plasma	6
Marek et al. [38]	2011	Poland	Cross-sectional	ELISA	108.36 ± 33.8	40.0 ± 15.32	54.93	26.0	10 (4)	0	10	Type 1	Serum	7
Mahdy and Nada [39]	2010	Egypt	Cohort	ELISA	44.90 ± 10.42	16.25 ± 2.05	60.52	na	20 (11)	10	10	Type 2	Serum	6
Zakareia et al. [40]	2010	Saudi Arabia	Cross-sectional	ELISA	200.30 ± 66.87	170.10 ± 7.45	na	na	30 (na)	0	30	Type 2	Plasma	6
Ozturk et al. [41]	2009	Turkey	Case-control	Others	173.59 ± 114.05	137.29 ± 84.45	62.09	na	95 (39)	49	46	Type 2	Serum	7
Skopiński et al. [42]	2007	Poland	Cross-sectional	ELISA	316.0 ± 412.91	445 ± 576.89	na	na	37 (na)	37	0	Type 2	Serum	3
Lee et al. [43]	2006	Korea	Case-control	ELISA	701.5 ± 400.9	508.0 ± 262.0	65.4	na	30 (16)	15	15	Type 2	Serum	6
Shimada et al. [44]	2002	Japan	Case-control	ELISA	25.0 ± 59.26	53.0 ± 82.96	na	na	30 (na)	na	na	Type 2	Plasma	3
Endo et al. [45]	2001	Japan	Case-control	ELISA	14.66 ± 18.36	25.66 ± 61.04	67.3	na	36 (na)	14	22	Type 2	Serum	5
Shinoda et al. [46]	1999	Japan	Case-control	ELISA	212.42 ± 15.66	173.0 ± 75.75	59.5	na	43 (na)	6	37	na	Serum	6
Burgos et al. [47]	1997	Spain	Case-control	ELISA	180.0 ± 120.0	210.0 ± 170.0	45.0	na	20 (na)	0	20	Type 1 & 2	Serum	4

VEGF: vascular endothelial growth factors; BMI: body mass index; DR: diabetic retinopathy; NPDR: nonproliferative diabetic retinopathy; PDR: proliferative diabetic retinopathy; ELISA: enzyme-linked immunosorbent assay; na: not available.

**Table 2:** Subgroup analysis of the included studies.

Subgroups	No. of studies	SMD (95% CI)	*P*	Test of heterogeneity	
				*I* ^2^	*P*
*Serum*					
Study location					
Asia	13	0.74 (0.42-1.07)	<0.001	91.3%	<0.001
Europe	5	0.39 (−0.20-0.98)	0.199	81.5%	<0.001
Africa	1	3.22 (2.06-4.38)	<0.001	/	/
Study design					
Cross-sectional	12	0.91 (0.54-1.29)	<0.001	92.5%	<0.001
Case-control	6	0.13 (−0.11-0.36)	0.283	25.0%	0.246
Cohort	1	3.22 (2.06-4.38)	<0.001	/	/

*Plasma*					
Study location					
Asia	7	0.09 (−0.14-0.31)	0.463	93.7%	<0.001
Europe	2	0.54 (−0.21-1.29)	0.156	0.0%	0.729
South America	1	−0.12 (−0.84-0.59)	0.734	/	/
Study design					
Cross-sectional	8	0.54 (−0.12-1.20)	0.108	91.7	<0.001
Case-control	1	−0.38 (−0.88-0.12)	0.557	/	/
Cohort	1	0.07 (−0.17-0.31)	0.133	/	/
